# A patient perspective on shared decision making in stage I non-small cell lung cancer: a mixed methods study

**DOI:** 10.1186/s12885-015-1974-6

**Published:** 2015-12-16

**Authors:** Wendy Hopmans, Olga C. Damman, Suresh Senan, Koen J. Hartemink, Egbert F. Smit, Danielle R. M. Timmermans

**Affiliations:** Department of Public and Occupational Health, EMGO+ Institute for Health and care research, VU University Medical Center, Van der Boechorststraat 7, 1081 BT Amsterdam, The Netherlands; Department of Radiation Oncology, VU University Medical Center, Amsterdam, The Netherlands; Department of Surgery, Netherlands Cancer Institute - Antoni van Leeuwenhoek Hospital, Amsterdam, The Netherlands; Department of Pulmonary Diseases, VU University Medical Center, Amsterdam, The Netherlands; Department of Thoracic Oncology, Netherlands Cancer Institute - Antoni van Leeuwenhoek Hospital, Amsterdam, The Netherlands

**Keywords:** Shared decision making, Stereotactic ablative radiotherapy, Surgery, Patient perspective

## Abstract

**Background:**

Surgery and stereotactic ablative radiotherapy (SABR) are both curative treatment options for patients with a stage I non-small cell lung cancer (NSCLC). Consequently, there is growing interest in studying the role of patients in treatment decision making. We studied how patients with stage I NSCLC perceived shared decision making (SDM) in general, and how they viewed different aspects of SDM.

**Methods:**

A sequential mixed methods design was used, consisting of qualitative interviews (*N* = 11), as well as a survey study (*N* = 76) focusing on different SDM-related aspects. Participants were interviewed to understand their own experience with treatment decision making. In the survey study, patients rated the importance of 20 aspects of shared decision making that were identified during interviews. Descriptive analysis and explorative factor analysis were performed.

**Results:**

We assessed six qualitative themes covering SDM aspects that were determined by patients to be important. The survey identified four SDM-related factors with sufficient internal consistency, namely (1) ‘guidance by clinician’ (α = .741), (2) ‘conduct of clinician’ (α = .774); (3) ‘preparation for treatment decision making’ (α = .864); and (4) ‘active role of patient in treatment decision making’ (α = .782). Of these, clinician guidance was rated as most important by patients (M = 3.61; SD = .44). Only 28.9 % of patients in the survey study reported that both treatment options were discussed with them.

**Conclusions:**

Patients with a stage I NSCLC found clinician guidance to be important when making treatment decisions. Nevertheless, the majority of patients reported not being offered both treatment options, which might have influenced this finding.

## Background

Shared decision making (SDM) by patients and clinicians is a process where clinicians and patients share the best available evidence and work together to select tests and treatments, and where patients are supported to consider options to achieve informed preferences [1, 2]. SDM has been increasingly accepted as a component of patient-centred high quality care [3, 4]. Studies in different health contexts indicate that patients prefer a SDM approach, and wish to be involved in treatment decisions [5, 6]. SDM can also improve a patient’s understanding of treatment options, increase confidence in the decisions made, and result in greater satisfaction with care provided [7]. SDM is also associated with improved treatment compliance and better quality of life [8], and it may reduce unwarranted medical practice variations [1, 9, 10], including the overuse of tests and elective procedures [11]. Consequently, SDM has been incorporated into the European Cancer Patient’s Bill of Rights [12], the Patient Protection and Affordable Care Act and the Salzburg Statement on Shared Decision Making [13].

Traditionally, SDM has been advocated for so-called ‘preference sensitive decisions’, which refer to situations where different, but equally effective, treatment options are available. In such cases, the ‘correct’ treatment depends on a given patient’s preference, specifically the relative weight a patient gives to the risks and benefits of treatment [14, 15]. In the field of oncology, it is increasingly recognized that patients’ preferences may depend on other aspects than only prolongation of life, for example on quality of life [16], which requires a reassessment as to how the decision making process can optimally be delivered [17].

A current decision problem in oncology is in the treatment of stage I non-small cell lung cancer (NSCLC), where two guideline-specified curative treatments are currently available, namely surgery and stereotactic ablative radiotherapy (or SABR) [18]. While surgery is considered the standard of care in medically operable patients, many patients are unfit to undergo surgery, or may decline to do so because of associated-risks. SABR is the preferred treatment for the latter group of patients [18]. The rapid growth in use of SABR for this patient population is because it is an outpatient technique that is associated with little high-grade toxicity, and long-term survivals after SABR appear similar to that reported for surgery [19]. Consequently, some clinicians consider the treatment of stage I NSCLC to be a preference sensitive decision, with equipoise between the two options. This, in turn, raises the question as to how a SDM process for this patient group should be delivered.

Implementing SDM in clinical practice can be challenging as patients may find participation difficult [7, 20], or may be ill-prepared for involvement in decision making [1]. Such issues may be more pronounced in older patients with cancer, who may have different expectations of the decision making process than what is currently being emphasized in SDM definitions. In general, patients appear to view SDM as a partnership between equals [21, 22], expect complete, honest and individualized information [23] and viewed SDM as a process that respected patient’s views about their health [21, 22, 24]. In contrast, some clinicians appear to view SDM as an approach to ensure that their patients comply with recommended treatments, in order to achieve good outcomes [21, 25]. Several studies have explored whether older cancer patients wish to be involved in treatment decision making, and the results have been conflicting. Some concluded that older cancer patients did prefer a more paternalistic approach, while others found that the elderly had a preference for SDM [5, 26, 27]. To the best of our knowledge, no studies have yet been reported on how patients with stage I NSCLC view specific aspects of SDM.

Several instruments have been developed and tested to assess how the treatment decision making processes actually occur in healthcare practice, including the observing patient involvement (OPTION) scale, the 9-item Shared Decision Making Questionnaire, the Facilitation of Patient Involvement in Care Scale, the Perceived Involvement in Care Scale, the Control Preference Scale and CollaboRATE [28–33]. Most of the former are patient-reported measures, and some are observational measures. Although patient perspectives are usually considered when developing such instruments, these instruments are generally not well-suited to assess the importance which patients attach to specific aspects.

The aim of our mixed methods study was to assess the views of patients with stage I NSCLC on aspects of SDM considered to be of greatest importance in the decision making process between surgery and SABR. In an initial interview study, we qualitatively examined how patients experienced the treatment decision making process, as well as factors which they found important. Subsequently, we quantitatively assessed the importance patients attached to different SDM aspects that had been identified in a survey study. In addition, we explored whether differences exist between subgroups of patients (for example younger (≤65) versus older patients (>65), males versus females, those with lower educational level versus those with medium and higher educational levels, lower health literacy levels versus those with higher health literacy levels, and those who reported that both treatment options were discussed versus those who did not) in their ratings of importance.

## Methods

### Study design

The prospective study was approved by the Medical Review Board of the VU University Medical Center (VUMC) and the Netherlands Cancer Institute - Antoni van Leeuwenhoek Hospital (NKI-AvL). Eligible participants had to have a diagnosis of stage I NSCLC no later than 2–6 months before inclusion, able to converse in the Dutch language, and provide written informed consent. Patients who underwent either surgery or SABR were eligible, irrespective of whether they had considered or rejected the alternative treatment.

A sequential mixed methods design was used [34], starting with a qualitative interview study conducted between February and April 2011. Next, the qualitative findings identified were used to design a survey for a larger patient population treated at the two institutions. Patients were identified using the institutional databases of the VUMC departments of Radiation Oncology and Surgery. The interviews focused on how patients experienced their own decision making process, and the factors which they found to be important in this process. The survey study was performed between October 2013 and May 2014, and eligible participants were recruited from the same departments. The survey captured data on aspects that patients had previously identified as being important in the qualitative interviews.

### Qualitative study

Sixteen eligible patients were invited by a postal letter to participate, and were subsequently contacted by telephone to assess their willingness to participate. Finally, 11 patients provided written informed consent, and in-person interviews were performed and audio-taped by the first author at the VUMC or at the patient’s home. For qualitative studies, it has been recommended that at least 8–15 participants be included to collect consistent data across individuals [35]. As no new topics emerged after nine interviews, our sample size was considered large enough to have reached saturation [36, 37]. The interviews were conducted with patients, jointly with relatives if present, using both an open and semi-structured approach.

In the open phase, we used a visual timeline to facilitate the process of telling stories about their healthcare trajectory and, more specifically, the treatment decision making process [38]. The interviewer (WH) instructed participants as follows: “This is a timeline. We are now at the end of your treatment process. I would like you to go back to the beginning, where it all started, when you heard your diagnosis and before a treatment decision was made. Can you recall and tell me about your experiences?” During the patients’ narrative of the decision making process, the interviewer made written notes of patients’ comments. The focus of the interviews emphasized two stages of SDM considered essential in the literature, intended to elicit patient perspectives on elements of these processes that patients considered essential: (1) the information collection process and (2) the decision making process. Examples of interview questions used were: “Can you tell me how the decision for surgery/SABR was made”; “Can you tell me how you experienced the process of making the decision?” Several socio-demographic characteristics were described to characterize the study population.

#### Data analysis

Analysis of the data involved the following steps [39, 40]: (1) The transcribed interviews were read and re-read, and key ideas on how patients experienced the decision making process were formed. (2) Two researchers (WH and OD) then independently analysed four transcripts, and both developed an initial list of codes. The two researchers held two consensus meetings to compare the codes and to categorize preliminary themes. (3) Additional transcripts were coded by the first author, and after consensus meetings, a coding tree was developed. This coding tree was composed of different subthemes related to how patients experienced the decision making process. (4) Sections were re-read to identify patients’ thoughts related to the decision making process. (5) The final stage involved a process of reflecting on the charted data to find associations between the themes, to provide explanations of the findings, and to synthesize the aspects that patients found important. Demographic data were summarized using descriptive statistics in SPSS for Windows, version 20.0 (SPSS Inc., Chicago, IL).

#### Quantitative study

A survey developed consisted of importance ratings of SDM aspects, in a four-point Likert scale from not important to very important, was identified from the qualitative study. These aspects were presented to patients as being ‘potentially important in the decision making process’. The survey also measured background characteristics of patients, namely age, sex, education, health literacy and the treatment which the patient had undergone. In addition, they were asked if more treatment options had been discussed by their treating clinician.

#### Data analysis

Explorative factor analysis (principal component analysis) with oblique rotation was performed on the survey items to determine whether factors could be formed representing stable aspects of SDM. We constructed composites, and calculated the internal consistency of these composites (Cronbach’s alpha). Descriptive analyses were conducted to assess the average importance scores on these composites. T-tests were performed to assess differences between subgroups of patients (younger (≤65) versus older patients (>65), males versus females, those with lower educational level versus those with medium and higher educational levels, lower health literacy levels versus those with higher health literacy levels, and those who reported both treatment options were discussed versus those who did not). All analyses were performed using SPSS for Windows version 20.0 [SPSS Inc., Chicago, IL).

## Results

Table 1 summarises the background characteristics of patients in both the qualitative and the quantitative study.

**Table 1:** Background characteristics of patients in qualitative and quantitative study

Patient characteristics		Number of patients (*n* = 11) Interviews	Number of patients (*N* = 76) Survey
		% (n)	% (n)
Age	<50	9 (1)	0 (0)
	50-64	18 (2)	17 (13)
	65-74	27 (3)	33 (25)
	≥75	46 (5)	46 (35)
	Missing	0 (0)	4 (3)
Sex	Male	46 (5)	62 (47)
	Female	54 (6)	36 (27)
	Missing	0 (0)	3 (2)
Education^a^	Low	36 (4)	37 (28)
	Medium	27 (3)	38 (29)
	High	36 (4)	24 (18)
	Missing	0 (0)	1 (1)
Health Literacy^b^	Low	27 (3)	54 (71)
	High	73 (8)	21 (28)
	Missing	0 (0)	1 (1)
Treatment	Surgery	45 (5)	22 (17)
	SABR	55 (6)	74 (56)
	Missing	0 (0)	4 (1)

^a^Low: primary school, lower level of secondary school or lower vocational training. Medium: higher level of secondary school, or intermediate vocational training. High: higher vocational training or university. ^b^Question “How confident are you filling out medical forms yourself” [56, 57]: Low health literacy: patients answered: some of the time, a little of the time or none of the time. High health literacy: patients answered: all of the time, most of the time

### Qualitative study

We identified six main themes representing aspects that patients found important in the decision making process about treatment options (Table 2). With regards to *the information collection process*, patients reported that provision of complete and detailed information was important, and that information about the disease itself and possible treatment options needed to be understandable (Theme 1: complete and understandable information). In addition, patients found it important to search for information themselves, e.g. on the Internet, and to ask questions to clinicians (Theme 2: active role of patients in information gathering). Furthermore, patients attached importance to hearing their clinician’s view on the treatment option in their situation (Theme 3: hearing preference of the clinician), particularly as one type of information source to take into account in decision making. Furthermore, a major theme was the professional approach by health professionals that was important to patients (Theme 4: conduct of professionals), with the affective aspects of being friendly, thoughtful and respectful, rated as most essential. Related to this, patients found it important that they were provided with the opportunity to express their own opinion and preferences about treatment options (Theme 5: opportunity to express own opinion). Finally, patients indicated that it was important that their family members had a role in the decision making process (Theme 6: role of family members), in particular in supporting the patient in decision making. Another finding of importance was that patients frequently mentioned that the choice between curative surgery and SABR had not been offered.

**Table 2:** Themes derived from the qualitative study

Information collection process	
Theme 1: Complete and understandable information *“I think it is important that a clinician is open to a patient. That they do not refrain from providing information. Nowadays, clinicians pay more attention to this than in the past. But clinicians are different in that. I’m lucky because my surgeon explains everything very thoroughly and in a straightforward way. I appreciate that ”. F, 69y* *“I think it is important that they < e.g. clinicians > explain everything in an understandable way. No fancy Latin names that patients do not understand and that results in saying to yourself when you are out of the office: I have cancer, but I don’t know what they are going to do. For me it is important that I understand everything”. M, 66y*	
Theme 2: Active role of patients in information gathering *“In advance, I made a list with questions I wanted to ask the clinician. Are there alternatives, is it an option to do nothing? I wrote 5 or 6 issues down. I came well-prepared” F, 66y* *“I read a lot on the Internet in that time period and I am pleased with that information and knowledge because I can actively take part in the conversation with the clinician” F, 66y*	
Theme 3: Hearing preference of the clinician *“Initially, they scheduled me for SABR, because of the fact that there was little tissue damage. During the second consultation with the clinician, I asked him, what would you advice your own father? Then he said: surgery”. M, 62y* *“And then my oncologist said to me, it is your decision, what do you want? Then I said, what do you recommend?”. M, 80y*	
Decision making process	
Theme 4: Conduct of professionals *“Many of the consultations with a clinician are technical. And they forget to feel compassion for a patient. There is no time to do that. When a clinician is very kind, that is 20 % of your recovery. Only being nice. And when he is disrespectful , you decline with 20 %”. F, 66y* *“In my opinion, it is important that a clinician is able to communicate with people, able to talk and to listen. And when you are not able to do that, you are a worthless clinician”. M, 78y*	
Theme 5: Opportunity to express own opinion *“The clinician wanted to operate immediately. I said, I want to think about that first. There’s also the option of radiotherapy” F, 69y* *“They < clinicians > wanted to operate me. I felt that my body wasn’t ready for another operation. Then I said that I wanted a second opinion. I wanted that very badly” F, 63y*	
Theme 6: Role of family members *“When it was clear what SABR was about and that it was 1 to 5 times, I clearly mentioned to my father, you can do that” Daughter of M, 78y* *“My husband joins me. We complement each other. We do all these things together. When there is something wrong with him I go along and when there is something wrong with myself, he goes along”. F, 80y*	

### Quantitative study

The survey assessed the main themes identified in the qualitative study, which were then translated into 20 survey items. We derived three items from theme 1, three items from theme 2, three items from theme 3, seven items from theme 4, three items from theme 5, and one item from theme 6 (Table 3). The disproportion in the numbers of items was related to the variety of specific aspects addressed by patients in discussing each theme. For example, patients mentioned a great variety of specific aspects related to professional conduct of clinicians, whereas they mainly addressed support during the decision making process when discussing the role of family members.

**Table 3:** Link between qualitative themes and quantitative items

Qualitative themes	Quantitative items
	Do you think it is important for your decision that …
Theme 1: Complete and understandable information	… you receive information from your clinician about all possible treatment options? … you receive information from your clinician about your disease? …your clinician gives you information about your disease that is understandable?
Theme 2: Active role of patients in information gathering	… you give your clinician information about how you experience your disease? … you search for information (for example on the Internet) about possible treatment options? … you ask the questions you have?
Theme 3: Hearing preference of the clinician	… you follow your clinician in the proposed treatment advice? … you decide together with your clinician about your treatment? … your clinician gives you advice about the best treatment option for you?
Theme 4: Conduct of professionals	*…* your clinician takes you seriously? … your clinician takes time for you? … your clinician is friendly? … your clinician asks you about your situation at home? … your clinician provides the opportunity to ask questions? … your clinician takes your treatment preferences seriously? … you receive time from your clinician to think about what treatment you want to have?
Theme 5: Opportunity to express own opinion	… your clinician asks you what you think of the different treatment options? … your clinician lets you decide what treatment you want to undergo? … the treatment that best fits for you is chosen?
Theme 6: Role of family members	… you eventually decide with your family what treatment you want to have?

In total, 76 of 150 (50.7 %) patients who were approached finally completed the survey, with response rates for patients who had undergone surgery being 22.4 % (*N* = 17), and for SABR patients 73.7 % (*N* = 56). Three respondents did not answer the question about the treatment which they had undergone. A total of 28.9 % of patients reported that both treatment options had been discussed by their clinician.

#### Factor analysis

Table 4 presents the details of the factor solution of all items and the resulting construction of composite measures. We identified four factors with both sufficient internal consistency and item clarity: (1) ‘guidance by the clinician’ (α = .741), (2) ‘conduct of clinician’ (α = .774); (3) ‘preparation for treatment decision making’ (α = .864); and (4) ‘active role of patient in treatment decision making’ (α = .782). Two items could not be included in these composite measures, namely ‘friendliness of the clinician’ and ‘you give your clinician information about how you experience your disease’. The factor loadings of these two items were low (<0.4) and the internal consistency of the composite measures did not improve when including them in these factors.

**Table 4:** Construction of scales with factor solutions and reliability analysis of 20 interview-based items

	Factor loading	ITC^a^	α if item deleted
Do you think it is important for your decision that…			
Construct 1: Guidance by the clinician (α = .741)			
… your clinician gives you advice about the best treatment option for you?	.850	.566	.656
… the treatment that best fits for you is chosen?	.562	.590	.646
… you ask the questions you have?	.528	.572	.669
Construct 2: Conduct of clinician (α = .774)			
*…* your clinician takes you seriously?	.866	.677	.683
… your clinician takes time for you?	.828	.723	.651
… your clinician takes your treatment preferences seriously?	.591	.479	.800
… your clinician provides opportunity to ask questions?	.444	.514	.751
Construct 3: Preparation for treatment decision making (α = .864)			
… you receive information from your clinician about all possible treatment options?	.847	.741	.827
… you receive information from your clinician about your disease?	.832	.584	.856
… you follow your clinician in the proposed treatment advice?	.781	.767	.823
… you decide together with your clinician about your treatment?	.761	.675	.839
… your clinician asks you what you think of the different treatment options?	.594	.643	.849
… your clinician gives you information about your disease that is understandable?	.539	.611	.852
Construct 4: Active role of patient in treatment decision making (α = .782)			
… you receive time from your clinician to think about what treatment you want to have?	.720	.706	.697
… you search for information (for example on the Internet) about possible treatment options?	.695	.398	.798
… you eventually decide with your family what treatment you want to have?	.665	.631	.715
… your clinician lets you decide what treatment you want to undergo?	.522	.539	.747
… your clinician asks you about your situation at home?	.494	.551	.744

^a^Item Total Correlation

The suitability of data for factor analysis was assessed by inspection of the correlation matrix, by computing the Kaiser-Meyer-Olkin measure of sampling adequacy value (KMO) and by running the Bartlett’s Test of Sphericity. KMO was .774 and Bartlett’s Test of Sphericity was < .00. KMO values of .60 or greater and a significant Bartlett’s Test of Sphericity for factor analysis were considered appropriate. An eigenvalue of 1.00 or greater was adopted as cut-off point to determine the number of components. An item’s factor loading of > .4 was used as a cut-off point for inclusion, followed reliability evaluation by calculations of Cronbach’s alpha

The mean importance scores on the composite measures and individual items are displayed in Table 5. Patients found ‘guidance by the clinician’ most important (M = 3.61; SD = .44). This composite covered items such ‘your clinician gives you advice about the best treatment option for you’ and ‘the treatment that best fits for you is chosen’. The factor ‘active role of patient in treatment decision making’ was reported to be least important (M = 2.75; SD = .71). No significant differences were observed between subgroups of patients (younger (≤65) versus older patients (>65), males versus females, those with lower educational level versus those with medium and higher educational levels, lower health literacy levels versus those with higher health literacy levels, and those who reported both treatment options were discussed versus those who did not) in their importance ratings of the constructs, nor for the individual items.

**Table 5:** Average importance scores of the 20 interview-based items

	M (SD)
Do you think it is important for your decision that…	
Construct 1: Guidance by the clinician (α=,741)	3.61 (.44)
… your clinician gives you advice about the best treatment option for you?	3.68
… the treatment that best fits for you is chosen?	3.71
… you ask the questions you have?	3.45
Construct 2: Conduct of clinician (α=,774)	3.53 (.46)
*…* your clinician takes you seriously?	3.68
… your clinician takes time for you?	3.64
… your clinician takes your treatment preferences seriously?	3.29
… your clinician gives space to ask questions?	3.51
Construct 3: Preparation for treatment decision making (α=,864)	3.46 (.49)
… you receive information from your clinician about all possible treatment options?	3.51
… you receive information from your clinician about your disease?	3.66
… you follow your clinician in the proposed treatment advice?	3.30
…you decide together with your clinician about your treatment?	3.51
… your clinician asks you what you think of the different treatment options?	3.14
…your clinician gives you information about your disease that is understandable?	3.63
Construct 4: Active role of patient in treatment decision making (α=,782)	2.75 (.71)
… you receive time from your clinician to think about what treatment you want to have?	3.05
… you search for information (for example on the Internet) about possible treatment options?	2.14
… you eventually decide with your family what treatment you want to have?	2.78
… your clinician let you decide what treatment you want to undergo?	2.88
… your clinician asks you about your situation at home?	2.91
Other items:	
… your clinician is friendly?	3.39
…you give your clinician information about how you experience your disease?	3.28

## Discussion

In this sequential mixed methods study examining how patients with stage I NSCLC perceive SDM, ‘guidance by the clinician’ was identified by patients as being the most important, and an active role by patients was considered relatively less important. A majority of patients (71.1 %) reported not being offered both treatment options (surgery and SABR), indicating that SDM was not taking place in many consultations.

Both qualitative and quantitative data revealed that patients considered the ‘expert’ advice from their clinician of great importance in the decision making process, a finding similar to that reported in other studies in patients with cancer [26, 27]. This finding is in contrast with formal definitions of SDM, which envisage a more active role for patients. The importance attached to guidance by clinicians may partly be accounted for by the fact that a majority of patients had no treatment options presented to them. If clinicians fail to have a ‘choice talk’ [41] with patients, in which it is explained that they are in a position to choose between treatment options, patients may end up attaching more importance to guidance by clinicians. A preference for a less active role might also be explained by a perceived lack of skills needed to participate in treatment decision making. For example, older patients have been found to have relatively more difficulties with processing information about treatment options [42]. In addition, more traditional attitudes (i.e., “the doctor knows best”) and lower motivation and ability to participate in decisions may be relevant factors [43–45].

Patients also considered the approach and conduct of the clinician (i.e., being emphatic/affective in communication), as being highly important in the context of treatment decision making. Affective communication aspects are recognized to be important to patients [46, 47], and our findings again emphasize the importance of supportive clinicians in the treatment decision making process. Although current definitions of SDM usually include supportive or ‘coaching’ elements, these seem to differ from the more guiding role that patients referred to in our study. For example, the International Patient Decision Aids Standards (IPDAS) criteria on coaching and guidance include assessing decisional needs, providing information, verifying understanding, clarifying preferences, building skills, screening for implementation needs, and facilitating progress in decision making [48]. These aspects do not directly cover conduct-related aspects. In clinical practice, the focus of SDM relies more on information provision from the clinician about alternative treatment options, with the patient giving and clarifying his or her preferences and values [41]. These SDM conceptions might not sufficiently take account of patients’ need for empathic communication styles of health professionals in making decisions.

Our finding that most patients were not provided with both options by their clinicians is consistent with a recent binary choice experiment among thoracic oncologists that showed that 45 % of thoracic oncologists did not consider surgery and SABR equal treatment options for stage I NSCLC patients, and that the patient’s preference did not heavily influence clinicians’ treatment recommendations [49]. The main reason for not considering both options to be equal was the perception that there is insufficient evidence, as no completed randomized controlled trials have been reported [49]. However, as the available comparative effectiveness data clearly suggest that SABR results in comparable outcomes to surgery [19, 50], and as guidelines [18] have taken this into account, more effort should be undertaken to increase awareness of this equipoise of options, for example during professional training of clinicians.

One limitation of our study was the relatively low response rate in the survey study. Although our number of respondents (*N* = 76) is considered acceptable for principal component analysis [51], some guidelines recommend higher numbers of respondents [52]. In addition, only 22 % of respondents had undergone surgery whereas 74 % were post-SABR patients, which might bias our findings. The reluctance of surgical patients to participate may, in part, be a reflection of the significant decline in quality of life following surgery in the post-surgical period, and in particular symptoms that persist in the first 6-months post-surgery [53]. In the Netherlands, fewer than 50 % of patients aged 70 years and older currently undergo surgery [54]. No differences were observed between patients who had undergone surgery and SABR patients in the importance attached to the SDM aspects, but the relatively small number of surgical patients should be noted. Our quantitative findings should also be interpreted in light of the fact that less than a third of patients described being offered both treatment options. Nevertheless, no significant differences were found between those who had been offered both options and those who had not been offered both options.

Our factor analysis revealed that four factors generally represented the dimensional structure of the survey in a stable way. However, ‘preparation for treatment decision making’ included two items (i.e. ‘your clinician asks you what you think of the different treatment options’ and ‘you follow your clinician in the proposed treatment’) on treatment discussion with the clinician that, when based on substantive arguments, might also suit to ‘guidance by the clinician’. Finally, recall bias could have been present as participants were included 2–6 months after hearing their diagnosis. Moreover, patient recall may also be influenced by their satisfaction with the treatment chosen and/or the healthcare process [55].

## Conclusions

Patients with a stage I NSCLC found guidance of clinicians, as well as their affective conduct, to be important in the treatment decision making process. Less than a third of our patients were in fact offered both treatment options, a finding which might have biased the observed findings. It appears necessary that current SDM conceptions and guidelines should address such guidance and clinician conduct more explicitly.

## Abbreviations

SABR: stereotactic ablative radiotherapy
NSCLC: non-small cell lung cancer
SDM: shared decision making
VUMC: VU University Medical Center
NKI-AvL: Netherlands Cancer Institute - Antoni van Leeuwenhoek Hospital
